# Doxorubicin-mediated testicular toxicity is associated with dysregulated mTOR/Beclin-1 pathways, oxidative stress, inflammation and apoptosis in Wistar rats: preventive role of lutein

**DOI:** 10.1186/s42826-025-00249-3

**Published:** 2025-06-09

**Authors:** Jennifer Efe Jaiyeoba-Ojigho, Jerome Ndudi Asiwe, Joseph Chimezie, Blessing Oluwakemi Abe, Oghenemarho Monalisa Ataikiru, Taniyohwo Mamerhi Enaohwo, Alexander Obidike Naiho, Lilian Ebele Chris-Ozoko, Godstime Jesukobiruo Ibada, Sylvester Ifeakachukwu Okuepusu, Favour Isioma Ikukaiwe, Winnie Taiye Ogwu

**Affiliations:** 1 Department of Anatomy, Faculty of Basic Medical Sciences, Delta State University, Abraka, Nigeria; 2 Department of Physiology, Faculty of Basic Medical Sciences, Delta State University, Abraka, Nigeria; 3 Department of Physiology, Faculty of Basic Medical Sciences, University of Ibadan, Ibadan, Nigeria; 4 Department of Anatomy, Faculty of Basic Medical Sciences, Lead City University, Ibadan, Nigeria; 5 Department of Physiology, Faculty of Basic Medical Sciences, University of Delta, Agbor, Nigeria

**Keywords:** Lutein, Doxorubicin, Steroidogenesis, Testicular toxicity, Autophagy

## Abstract

**Background:**

Lutein offers undoubted hope for preventing doxorubicin uncharacteristic reproductive function which remains a worldwide health concern in cancer chemotherapy. However, the mechanisms underlying the effect of lutein on maintaining the male reproductive milieu have not yet been fully identified. The current study investigates the preventive effect of lutein in doxorubicin-induced testicular toxicity.

**Methods:**

Twenty male Wistar rats were randomly assigned into four groups of five animals (*n* = 5) and were pretreated with lutein (40 mg/kg i.p) prior to doxorubicin treatment (15 mg/kg i.p). Following the end of the experiment, animals were euthanized and the testes were collected and processed for semen and biochemical analysis.

**Results:**

The results revealed that exposure to doxorubicin caused hormonal imbalance, reduced semen quality and elicited oxidative stress, inflammatory reactions, apoptosis as well as dysregulation of autophagic process which was accompanied by fibrosis and histomorphological aberrations. Interestingly, pretreatment with lutein significantly restored hormonal balance and protected against the adverse effects of doxorubicin.

**Conclusions:**

The findings of this study showed that lutein prevents doxorubicin-mediated testicular toxicity via modulation autophagic pathways accompanied with inhibition of oxidative stress, inflammation and apoptosis.

## Background

Male fertility is negatively impacted by a number of factors, including chemical usage, smoking, radiation, infections, varicocele, psychological stress, epigenetic changes, exposure to environmental pollutants and mitochondrial dysfunction [1]. In cancer therapies, doxorubicin, also referred to as anthracycline, is a well-known anti-tumor drug. A significant worldwide health concern is the association between doxorubicin therapy and subfertility and abnormal reproductive health [2]. Doxorubicin (DOX) has been linked to abnormal reproductive health, according to Chen et al. [3] and Yun et al. [4], through the release of inflammatory mediators, cellular apoptosis, changes in cholesterol synthesis, inhibition of DNA replication through the alpha topoisomerase II isoform and production of reactive oxygen and nitrogen species. The disruption of steroidogenic enzyme activity caused by doxorubicin, including 17α-hydroxylase and 3β-hydroxysteroid dehydrogenase (3β-HSD), is noteworthy as it impacts hormones related to the hypothalamic-pituitary-gonadal (HPG) axis, including luteinizing hormone, testosterone and follicle-stimulating hormone [5].

The correlation between DOX and multi-organ toxicity has also been linked to dysregulated autophagic mechanisms [6, 7]. Regulating mTOR/beclin-1 activity is one possible therapy option to mitigate the deleterious effects of DOX, as there is mounting evidence that it may have a direct impact on spermatogenesis and conception [8]. Faulty autophagy genes are also linked to apoptosis, anomalies in mitochondrial activity and oxidative stress in the testicles. Moreira et al. [9] have also noted the maintenance and remodelling of the blood-testis barrier (BTB), which is crucial to the seminiferous tubule epithelial activities. However, there’s evidence that overexpression of beclin-1 is linked to testicular damage [7].

Many have reported that diets high in broccoli, lettuce, turnip greens, almonds, spinach, peas and egg yolk are good sources of lutein, a kind of xanthophyll, an oxygenated carotenoid [10–14]. Anti-inflammatory, antioxidant and anti-apoptotic properties of lutein have been linked to its beneficial benefits in previous studies [12–14]. The ability of lutein to treat a wide range of conditions, including obesity, intestinal diseases, neurological disorders, abnormalities of the eyes, cardiovascular diseases, skin diseases, and liver injuries, has been shown to have significant therapeutic effects [12, 15, 16]. As mentioned by Oyovwi et al. [14], lutein has shown its potential therapeutic efficacy against reproductive dysfunction by downregulating NOX-1 signalling and upregulating Nrf2/HO-1/Cx-43 signalling. However, no data exists to support the idea that lutein could protect against doxorubicin-induced testicular impairment. The current study investigates the mechanism by which lutein shields Wistar rats against doxorubicin-induced testicular damage.

## Methods

### Compounds

AK Scientific, Inc., located at 30,023 Ahern Avenue, Union City, CA 94,587, USA, provided the doxorubicin (25316-40-9) utilized in this study. Sigma Aldrich, located in St. Louis, MO, USA, supplied lutein. Elabscience Biotechnology Inc. (USA) provided the following ELISA kit products: 3-β-HSD, Beclin-1 (E-EL-H0564), mTOR (ab206311), TNF-alpha (E-EL-R2856), IL6 (E-EL-R0015), Caspase 3 (E-EL-R0160), Bcl-2 (B-cell Lymphoma/Leukemia 2; E-EL-R0096), and Testosterone (E-EL-0155), FSH (E-EL-R0391), and LH (E-EL-R0026). Analytical grades applied to every other material.

### Animal procurement and experimental design

Twenty (20) male Wistar rats weighing 180 ± 10 g was used in this experiment. They were purchased from the animal breeding facility of the Faculty of Basic Medical Sciences at Delta State University and housed in the animal house of the Department of Physiology. In a well-ventilated plastic cage with free access to food and water, the animals were first given time to adjust to the temperature and light and shade of a laboratory setting, which lasted for 7 days. The approval was granted by the Delta State University animal ethics committee (RBC/FBMS/DELSU/24/302). The animals were divided into four groups at random, each consisting of five rats (*n* = 5) and given different treatments. The control group received 10 ml/kg body weight of distilled water; the DOX group received 15 milligrams of doxorubicin [16]; the Lutein (LUT) group received 40 mg/kg of lutein [16]; and the LUT + DOX treatment group received 40 mg/kg of lutein plus 15 mg/kg of doxorubicin. Groups 2 of the experimental rats received a 3-day injection of 15 mg/kg of doxorubicin, while groups 3 and 4 of the rats received a 25-day pretreatment with lutein in order to generate a preventive experimental model. For every therapy process, peritoneal injections were utilized. The present study drug dosage and protocol were adapted from our previous studies that reported the preventive impact of lutein on doxorubicin-mediated liver damage [16]. A 24-hour period after the last day of the experimental phase saw the animals put to death while they fasted during the night. Testicular and serum samples were used for biochemical analysis as well as histological inspection.

### Hormonal profile

Serum levels of luteinizing hormone (LH), testosterone and follicle stimulating hormone (FSH) were measured after centrifuging blood samples for 15 min at 3000 rpm. To measure the serum levels of FSH, LH and testosterone, ELISA kits (Elabscience Biotechnology Inc. (USA)) were used following the manufacturer’s instructions.

### Measurement of semen volume

Semen volume was measured using approximation method as described by Oyovwi et al. [14]. Briefly, normal saline was added to liquefy the semen. Then the volume of diluent added represents the approximate volume of the semen. This method assumes that the semen is entirely suspended and homogenised in the diluent, allowing indirect volume estimation.

### Sperm count

Using the methods presented by Raji et al. [17], the number of sperm was determined. After the left cauda epididymis was removed, it was placed in 2 mL of sterile saline solution (0.9% sodium chloride) and pre-warmed to 37ºC. Following a series of tiny punctures in the tissue to remove the sperm from the cauda epididymis, we used a Pasteur pipette to transfer the 200 µL sperm-saline suspension into the chambers of the modified Neubauer hemocytometer. Sperm count and quantity were measured in millions per milliliter of solution using a Leica DM 750 microscope.

### Sperm morphology

Sperm morphology was examined using the Narayana et al. [18] methodology. In a nutshell, a drop of the sperm suspension was applied to the glass slide and allowed to air dry. The picture was then enlarged 400 times using a Leica DM 750 light microscope. For every sperm that was tested, the amounts of anomalies in the head, middle and tail were added together.

### Sperm motility

Sperm motility was measured using the methodology of Atashfaraz et al. [19]. A prewarmed glass slide containing 10 µL of sperm suspension was examined using an X100 magnification Leica DM 750 light microscope on a heated stage (37ºC).

### Tissue Preparation

The testes were homogenized in phosphate-buffered saline, per the earlier description provided by Asiwe and colleagues [20]. The homogenates were then placed in a cold centrifuge (-4ºC) and spun for 15 min at 3000 rpm. In the supernatants, the levels and activities of proinflammatory cytokines, antioxidants and testicular enzymes were assessed.

### B-cell lymphoma factor-2

As instructed by the manufacturer, the ELISA method was utilized to determine the levels of B-cell lymphoma factor-2 (Bcl-2) in the testes. After they were all mixed together, 100 µL of the sample or standard was put into each well. During incubation, this was done for ninety minutes at 37 °C. A 100 µL biotinylated detection Ab working solution was added to each well and allowed to sit at 37 °C for 60 min. The solutions were removed from the plate after three washing cycles. The mixture required 30 min of 37 °C incubation after 100 µL of HRP conjugate working solution was added. Furthermore, five washings were performed on the solution. A microplate reader was used to measure the absorbance at 450 nm after 50 µL of stop solution was applied and 90 µL of substrate reagent was incubated for 15 min at 37 °C.

### Caspase-3 activities

To identify caspase-3 activity, the enzyme must hydrolyse the peptide substrate, acetyl-Asp-Glu-Val-Asp p-nitroanilide. P-nitroaniline is tested for concentration using spectrophotometry at 405 nm wavelength when it is discharged from the procedure. The procedure for assessing caspase-3 activity was carried out in compliance with the manufacturer’s guidelines.

### Testicular Oxido-nitrosative stress

Using spectrophotometric technique, Habig et al. [21] determined the amount of GSH-CDNB conjugate by measuring glutathione-S-transferase (GST) expressed in minutes per milligram protein (U/mg protein). The testis’ GSH (µM/mg protein) was measured in accordance with Jollow et al. [22] earlier description. Furthermore, as per previous publications by Misra and Fridovich [23] and Claiborne [24], respectively, the levels of catalase (U/mg protein) and superoxide dismutase (SOD) (U/mg protein) were measured. Meanwhile, the thiobarbituric acid reaction [25] was used to test malondialdehyde (MDA) expressed as µM/mg protein and the Griess reagent was used to measure nitrite at µM/mg protein [26].

### Measurement of Beclin-1, mTOR, and pro-inflammatory cytokines in the testicles

Using the corresponding ELISA kits, testicular TNF-α and IL-6 expressed as pg/mg protein, as well as mTOR and Beclin-1 expressed as ng/mL, were assessed, as per the manufacturer’s instructions.

### Histopathology of the testicles

The left testis was kept in Bouin’s solution for seventy-two hours, in accordance with the earlier research of Kolawole et al. [27], and Asiwe et al. [16],. An extractable 5-µm sample of the testis was subjected to the H&E staining to reveal the histomorphological changes microscopic capturing at x400 magnification while Masson trichome technique was used to determine, collagen and extracellular matrix deposition. Following staining with mason trichrome, cytoplasm stained red, nucleus stained black while the fibrotic area stained blue. The slides were captured with a microscope at x200 magnification and the Image-J deconvolution plugin were used to calculate the average of three separate sections per animal’s proportion of the testicular fibrotic region.

### Statistics

After the data were analysed with Graph Pad Prism (9.5), the mean ± standard error mean (SEM) was displayed. The Tukey multiple comparison test was used after a one-way analysis of variance (ANOVA) was utilized to ascertain the group differences. Significant results were defined as *P* < 0.05.

## Results

### Lutein prevents DOX-induced dysregulation of hormones and steroidogenesis

**Fig. 1:**
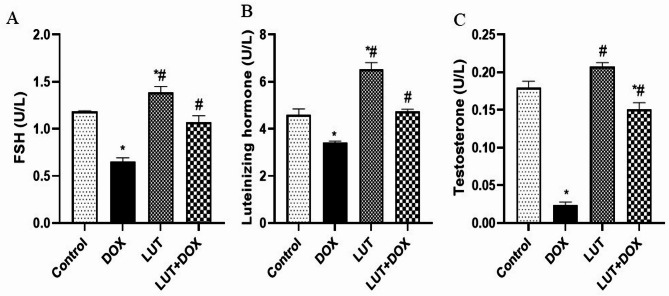
Lutein prevents DOX-induced dysregulation of hormones and steroidogenesis. (**A**) Follicle stimulating hormone (FSH) (**B**) Luteinizing hormone (LH) (**C**) Testosterone. Data expressed as mean ± SEM, *n* = 5. Analysis by one-way ANOVA followed by Tukey post-hoc test. * *p* < 0.05, versus Control. # *p* < 0.05, versus DOX

**Fig. 2:**
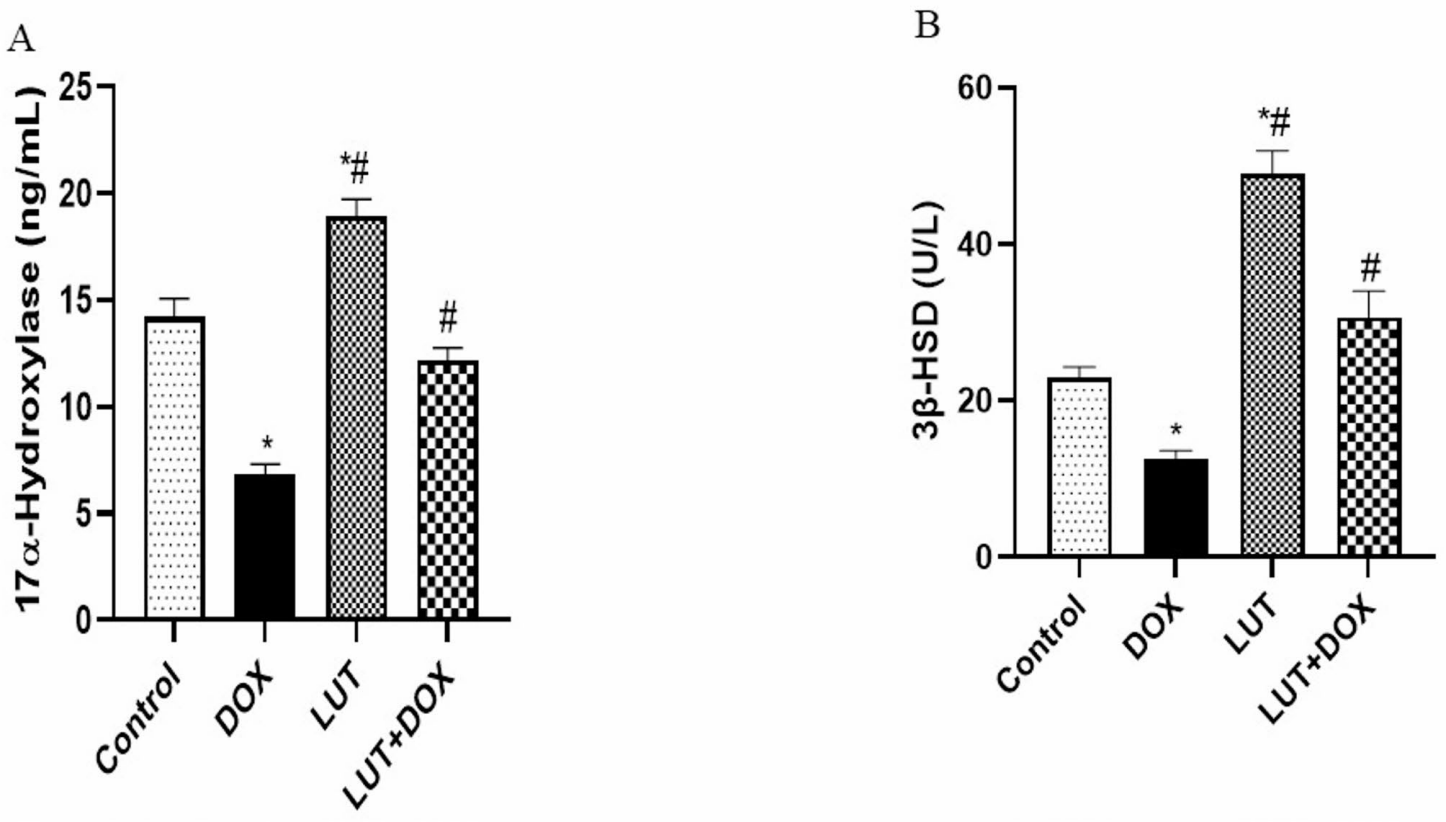
Lutein prevents DOX-induced steroidogenesis dysregulation. (**A**) 17α-hydroxylase (**B**) 3β-Hydroxyl steroid dehydrogenase (3β-HSD). Data expressed as mean ± SEM of *n* = 5. Analysis by one-way ANOVA followed by Tukey post-hoc test. * *p* < 0.05, versus Control. # *p* < 0.05, versus DOX. LUT = Lutein while DOX = Doxorubicin

The levels of FSH, LH, and testosterone in lutein-pretreated rats are illustrated in Fig. 1A-C. DOX-group observed a decrease in FSH (F (3, 12) = 34.98, *P* < 0.0001), LH (F (3, 12) = 41.78, *P* < 0.0001) and testosterone (F (3, 12) = 139.1, *P* < 0.0001) when compared to the control group. On the other hand, pretreatment with lutein increased the levels of FSH, LH, and testosterone when compared to DOX-exposed group. Similarly, Fig. 2A-B also showed that, the level of steroidogenic enzymes including 17α-Hydroxylase (F (3, 12) = 50.49, *P* < 0.0001) and 3β-HSD (F (3, 12) = 40.26, *P* < 0.0001) levels were significantly decreased in DOX-group relative to control. In contrast, LUT-pretreated rats prevented the effect of DOX on the testicular levels of 17α-Hydroxylase and 3β-HSD enzymes.

### Lutein protects against DOX-induces sperm abnormalities

**Fig. 3:**
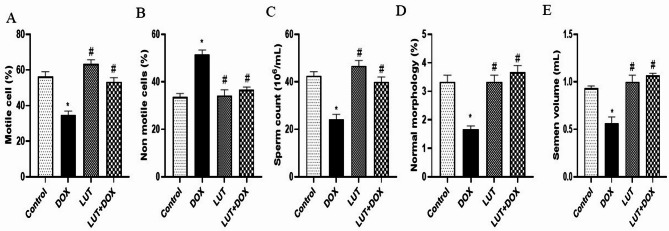
Lutein protects against DOX-induces sperm abnormalities (**A**) motile cell (**B**) non-motile cell, (**C**) Sperm count. (**D**) Normal morphology (**E**) Semen volume. Data expressed as mean ± SEM of *n* = 5. Analysis by one-way ANOVA followed by Tukey post-hoc test. * *p* < 0.05, versus Control. # *p* < 0.05, versus DOX. LUT = Lutein while DOX = Doxorubicin

In Fig. 3A-E, DOX group was observed to significantly increase the percentage non-motile sperm cells (F (3, 12) = 25.80, *P* < 0.0001) while it decreased percentage motile sperm cells [F (3, 12) = 22.05, *P* < 0.0001], sperm count [F (3, 12) = 22.29, *P* < 0.0001], normal morphology [F (3, 12) = 18.05, *P* < 0.0001] and semen volume [F (3, 12) = 19.96, *P* < 0.0001] when compared with control. However, Lutein prevented the effect of DOX by increasing significantly the percentage motile sperm cells, sperm count, normal morphology and semen volume as well as a decreased in non-motile sperm cells when compared with DOX-treated group.

### Lutein averted DOX-induced testicular oxidative stress

**Fig. 4:**
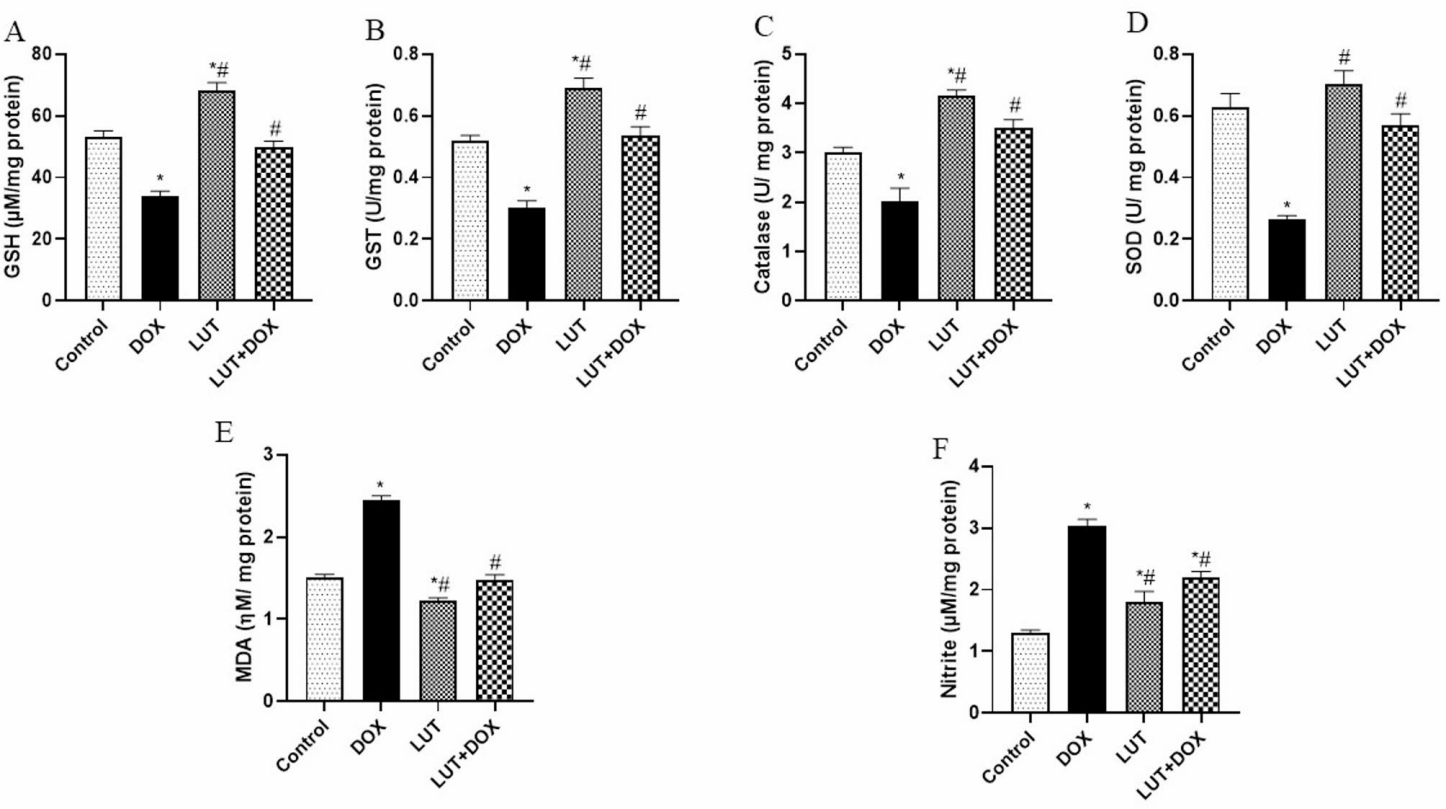
Lutein averted DOX-induced testicular oxidative stress. (**A**) Glutathione (GSH) (**B**) Glutathione s-transferase (GST) (**C**) Catalase (**D**) Superoxide dismutase (SOD) (**E**) Malondialdehyde (MDA) (**F**) Nitrite. Data expressed as mean ± SEM of *n* = 5. Analysis by one-way ANOVA followed by Tukey post-hoc test. * *p* < 0.05, versus Control. # *p* < 0.05, versus DOX. LUT = Lutein while DOX = Doxorubicin

As demonstrated in Fig. 4A-F. DOX group observed a significant testicular oxidative stress by decreasing GSH [F (3, 12) = 49.57, *P* < 0.0001] level, GST [F (3, 12) = 37.36, *P* < 0.0001], Catalase [F (3, 12) = 27.19, *P* < 0.0001] and SOD [F (3, 12) = 27.99, *P* < 0.0001] activities while MDA [F (3, 12) = 120.4, *P* < 0.0001] and nitrite [F (3, 12) = 41.93, *P* < 0.0001] levels were increased significantly when compared to control. Conversely, the LUT-pretreated group observed a significant increase in testicular antioxidant activity including GSH, GST, Catalase and SOD with a corresponding significant decrease in MDA and nitrite levels when compared to the DOX group.

### Lutein pretreatment suppressed DOX-induced inflammatory reactions

**Fig. 5:**
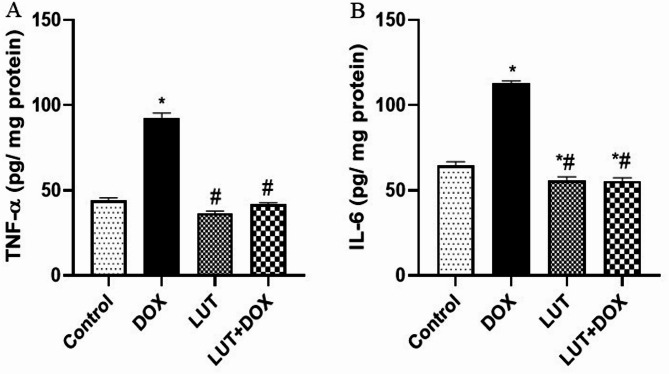
Lutein modulates DOX-induced inflammatory cytokines. (**A**) Tumor necrosis factor-alpha (TNF-α) (**B**) Interleukin 6 (IL-6). Data expressed as mean ± SEM of *n* = 5. Analysis by one-way ANOVA followed by Tukey post-hoc test. * *p* < 0.05, versus Control. # *p* < 0.05, versus DOX. LUT = Lutein while DOX = Doxorubicin

As shown in Fig. 5A-B, experimental rats treated with DOX only observed a significant increase in testicular TNF-α [F (3, 12) = 221.1, *P* < 0.0001] and IL-6 [F (3, 12) = 217.7, *P* < 0.0001] relative to control. However, lutein pretreated rat suppressed DOX-mediated testicular TNF-α and IL-6 release.

### Lutein downregulates DOX-induced apoptotic process

**Fig. 6:**
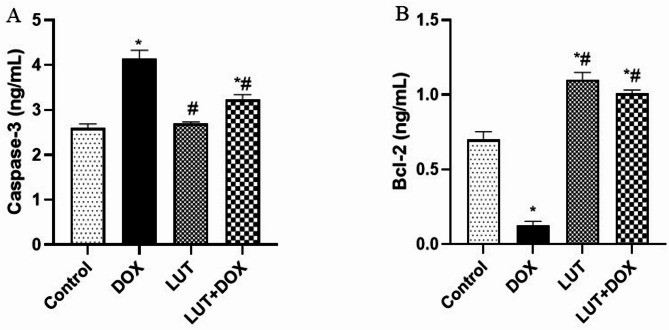
Lutein downregulates DOX-induced apoptotic process. (**A**) caspase-3 (**B**) B-cell lymphoma factor 2 (Bcl-2). Data expressed as mean ± SEM of *n* = 5. Analysis by one-way ANOVA followed by Tukey post-hoc test. * *p* < 0.05, versus Control. # *p* < 0.05, versus DOX. LUT = Lutein while DOX = Doxorubicin

Experimental rats treated with DOX only observed a significant increase in testicular caspase-3 [F (3, 12) = 35.74, *P* < 0.000] and a decrease in Bcl-2 [F (3, 12) = 113.6, *P* < 0.000] compared to control. Meanwhile, lutein-pretreated rats prevented DOX effects on testicular caspase-3 and Bcl-2 (Fig. 6A-B).

#### Lutein prevent DOX-induced dysregulation of testicular autophagy signalling

**Fig. 7:**
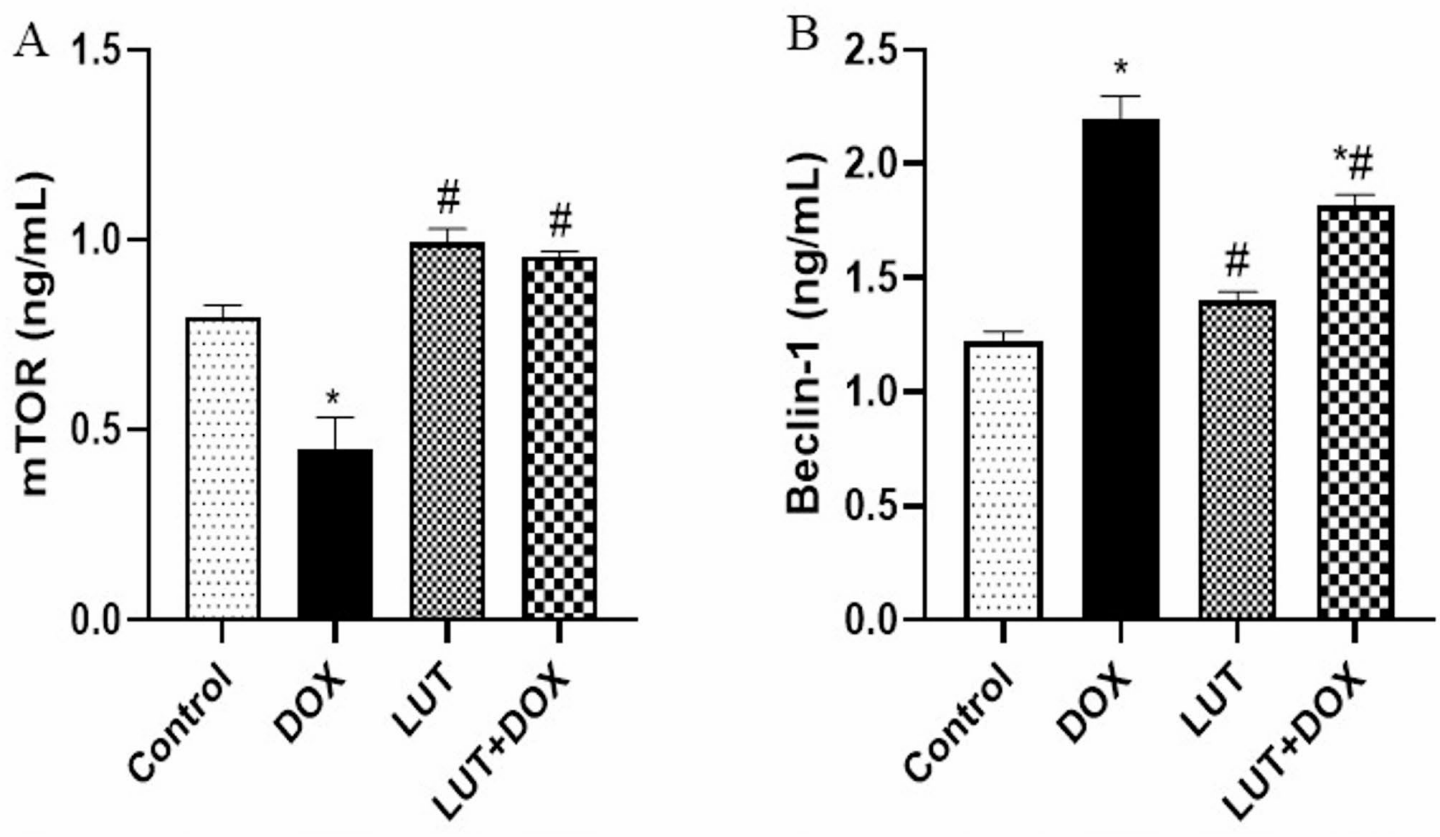
Lutein prevent DOX-induced dysregulation of testicular autophagy signaling (**A**) mammalian target of rapamycin (mTOR) (**B**) Beclin-1. Data expressed as mean ± SEM of *n* = 5. Analysis by one-way ANOVA followed by Tukey post-hoc test. * *p* < 0.05, versus Control. # *p* < 0.05, versus DOX. LUT = Lutein while DOX = Doxorubicin

As presented in Fig. 7A-B, experimental rats treated with DOX only observed a significant decrease in testicular mTOR [F (3, 12) = 27.66, *P* < 0.0001] and an increase in Beclin-1 [F (3, 12) = 48.43, *P* < 0.0001] compared to the control. Meanwhile, lutein-pretreated rats prevented DOX effects on testicular mTOR and Beclin-1.

### Lutein prevented abnormal testicular histopathology

**Fig. 8:**
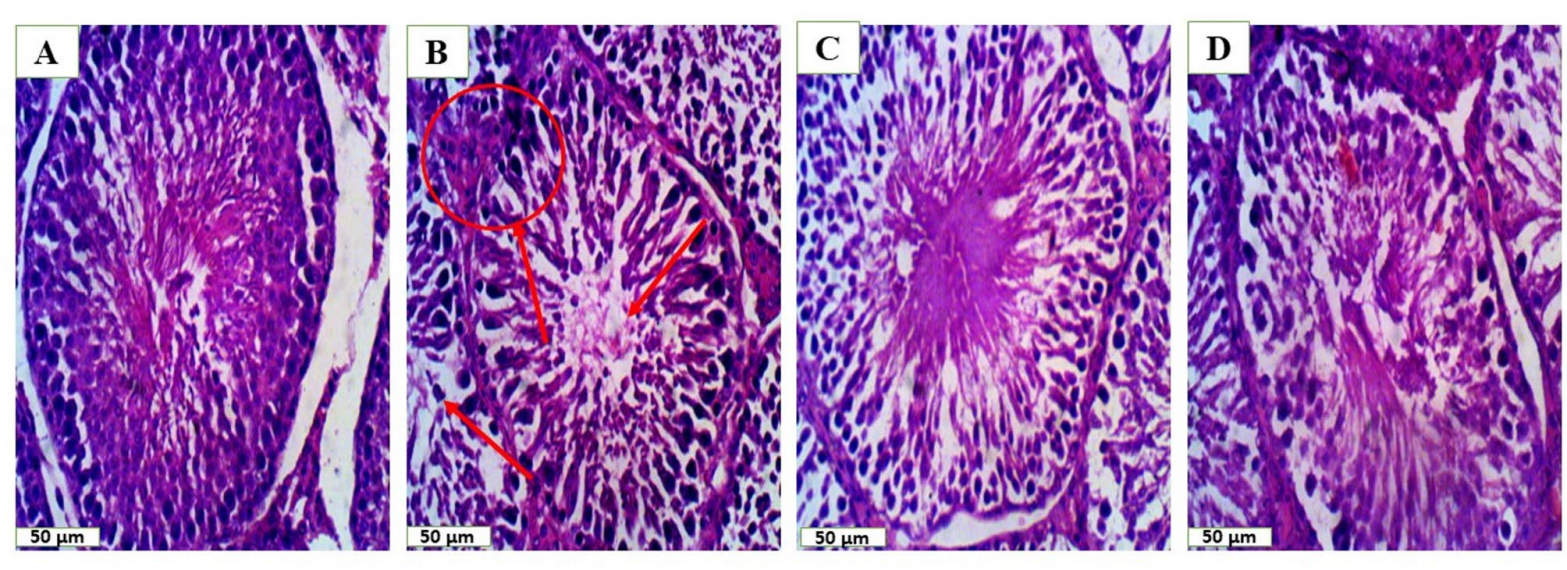
Lutein prevented abnormal testicular histopathology. (**A**) Control (**B**) DOX (**C**) LUT (**D**) LUT + DOX. Red arrows indicate areas of significant lesion. The slide was stained with H&E and photographed with microscope at X400 magnification. LUT = Lutein while DOX = Doxorubicin

**Fig. 9:**
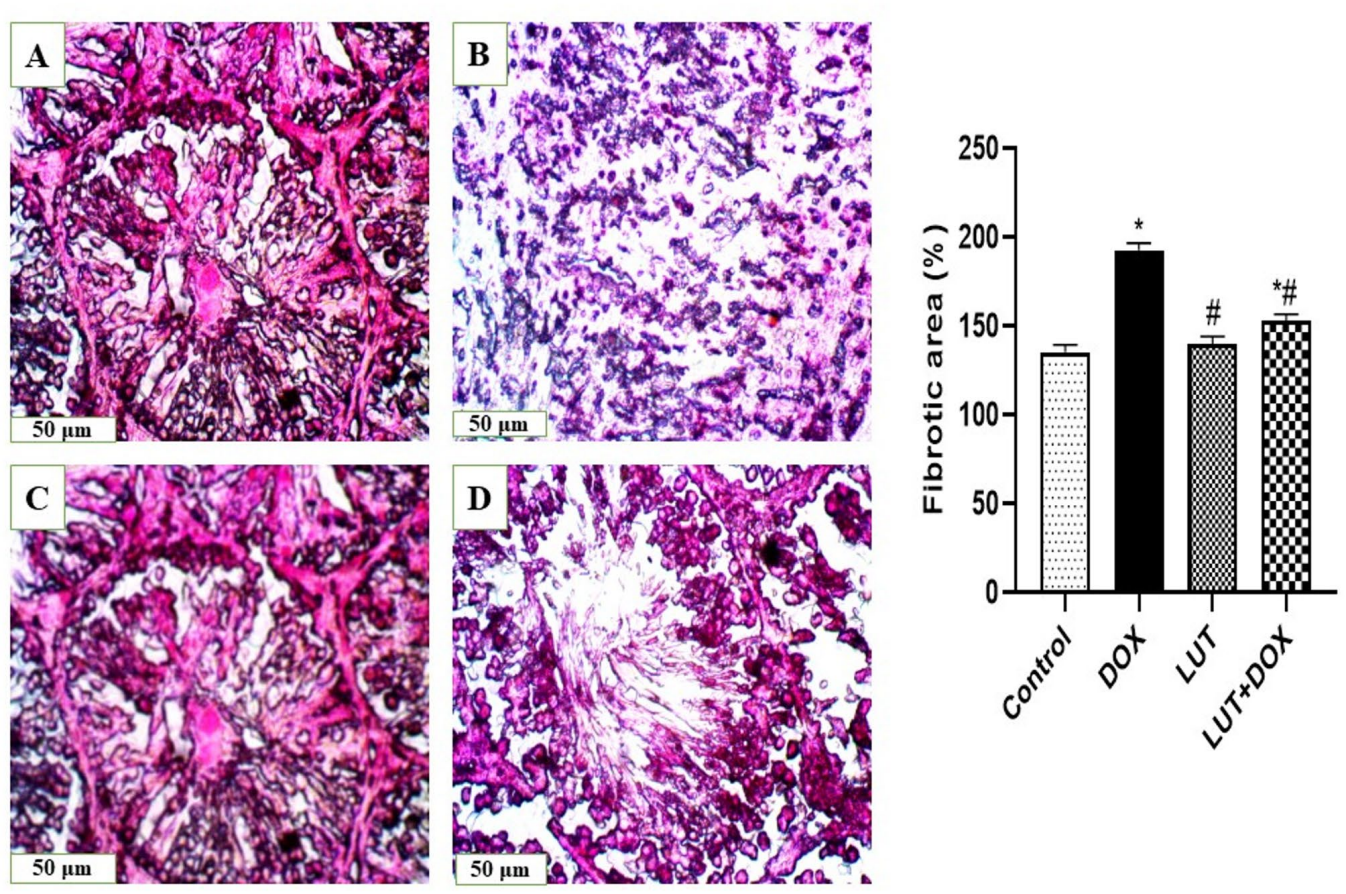
Lutein abates testicular fibrosis **A**) Control (**B**) DOX (**C**) LUT (**D**) LUT + DOX. Tissues were stained with mason trichrome and snapped at X400 magnification. Data expressed as mean ± SEM of *n* = 5. Analysis by one-way ANOVA followed by Tukey post-hoc test. * *p* < 0.05, versus Control. # *p* < 0.05, versus DOX. LUT = Lutein while DOX = Doxorubicin

Experimental rats treated with DOX-only demonstrated severe impairment of spermatogenic activity, marked by spermatogenic germ cell arrest including substantial damage to the seminiferous epithelium, the emergence of vacuoles, and an uneven distribution of spermatogenic cells compared with control (Fig. 8). Also, DOX-only treated rats showed a significant increase in collagen deposition leading to increased fibrotic area [F (3, 12) = 40.68, *P* < 0.0001] relative to the control (Fig. 9). In contrast, lutein-pretreated rats prevented DOX-induced testicular necrotic neoplasm, spermatogenic germ cell arrest and seminiferous epithelium degeneration as well as reduced collagen deposition resulting in reduced fibrotic area.

## Discussion

It has been established that doxorubicin-mediated testicular toxicity is caused by a variety of pathologic signalling pathways, including dysregulated autophagic processes, oxidative stress, inflammation and apoptosis as well as aberrant sperm and testicular morphology [28]. This study investigated lutein’s capacity to shield testicles against doxorubicin-induced damage. It’s interesting to note that lutein protected against these aberrant functions of the testicles, as evidenced by enhanced antioxidant, anti-inflammatory and anti-apoptotic activities in the testicles; additionally, the restoration of dysregulated beclin-1/mTOR signalling was accompanied by improved histomorphology and the suppression of fibrotic plaque formation.

Using a variety of parameters, such as sperm analysis, oxidative stress, pro-inflammatory makers, apoptotic makers, autophagic biomarker and steroidogenic enzymes (3β-hydroxysteroid dehydrogenase (3β-HSD) and 17α-hydroxylase), this study offers significant insights into the protective effect of lutein against doxorubicin-induced reproductive toxicity in rats.

Our results showed that DOX significantly decreased FSH, LH, and testosterone, which is consistent with previous research showing hormonal imbalance and infertility [5, 14, 28, 29]. Notably, appropriate leydig cell physiology and the maintenance of Sertoli cell activity throughout spermatogenesis depend on the balance between FSH and LH, a secretion stimulated by gonadotropin releasing hormone (GnRH). Our results suggest that a negative feedback loop affecting the synthesis of testosterone in Leydig cells may have resulted from the pituitary-testicular hormone imbalance [30]. Therefore, infertility in men is mostly caused by an imbalance in male reproductive hormones. Given that the underlying mechanism causing the DOX-induced imbalance in reproductive hormones includes levels of steroidogenic enzyme activity, we looked at how lutein affected the activities of 3β-HSD and 17α-Hydroxylase. In line with previous studies [5, 14, 29], our results showed that rats exposed to DOX had reduced levels of 3β-HSD and 17α-Hydroxylase. It is noteworthy that 3β-HSD is necessary for the synthesis of every steroid hormone. According to studies, the loss of 3β-HSD regulatory actions may lead to the testes producing less testosterone, which may induce sperm cell degeneration and possibly seminal tubule distortion [31–34]. Consistent with other research, rats administered DOX exhibited a notable decrease in the number of motile sperm cells, sperm count, normal morphology and semen volume, along with an increase in the proportion of non-motile sperm cells [14, 29]. Fascinatingly, our findings demonstrated that lutein stopped the DOX-induced reduction in non-motile cells, sperm count, normal morphology, and semen volume. Interestingly, lutein provided defense against DOX-induced cellular damage by reestablishing hormone balance and steroidogenic enzyme activity.

In this investigation, DOX-treated experimental rats displayed elevated testicular lipid peroxidation and nitrosative stress along with decreased testicular antioxidant activity (GSH, GST, SOD, and CAT). This implied that the production of reactive oxygen species and nitrogen species was markedly increased. Rats given lutein, however, showed a considerable boost in antioxidant activity by strengthening the body’s natural defenses against antibodies, as shown by increased levels of testicular SOD, GSH, GST and catalase. According to Ahn et al. [11], these findings imply that dangerous testicular oxidative by-products such as singlet oxygen, lipid peroxyl radicals, hydroxyl radicals (OH^−^), and superoxide anions (O_2_^−^) may give rise to hydrogen peroxide (H_2_O_2_), which is then converted into water. Further research has linked increased nitrite (NO) to cytotoxicity resulting in ischemia, neurodegenerative illnesses and inflammation in addition to other pathological processes [35, 36]. Given that the rats were administered DOX, their testicles displayed elevated levels of MDA and NO, which indicated nitrosative and oxidative stress. Lower levels of MDA and NO in the testicles, however, show that lutein mitigated this damage.

Dutta et al. [37] and Hasan et al. [38] have reported that doxorubicin-induced testicular toxicity is caused by pathobiological mechanisms that include higher levels of proinflammatory cytokines and activation of apoptotic signalling pathways. However, lutein was found to suppress the apoptotic and inflammatory responses, as seen by increased Bcl-2 and lower levels of TNF-α, IL-6, and caspase 3. This was in contrast to the group exposed to DOX. Notably, and in agreement with previous studies, it has been demonstrated that Bcl-2 protein activators and caspase 3 protein inhibitors may enhance cell survival in pathogenic conditions [39, 40].

Recent investigations demonstrated that lutein has a comparable effect on autophagic signalling proteins. Our observations are consistent with earlier studies [14, 41] showing that rats exposed to DOX exhibited downregulated testicular mammalian target of rapamycin (mTOR) and increased Beclin-1 activity. The regulation of growth factor and nutrition signalling, as well as the redox balance, is linked to the activity of the mammalian target of rapamycin (mTOR) [42]. Because sertoli cells are essential for the nutritional support of spermatogenesis, the involvement of growth factor and nutrient signalling mTOR in the maintenance and differentiation of spermatogonia stem cells is noteworthy [41–43]. Reduced testicular function, along with elevated proinflammatory mediators and testicular oxidative stress, leads to sperm DNA damage and apoptosis, lipid peroxidation, protein oxidation, mTOR inhibition and elevated Beclin-1 signalling. These occurrences, damage the blood-testis barrier, sperm plasma membrane and DNA integrity, which leads to a disruption in spermatogenesis, according to Sabeti et al. [44] and Dutta et al. [37]. However, the favorable therapeutic effects of lutein remarkably counteract the negative consequences of DOX.

The anti-apoptotic, anti-inflammatory and antioxidant properties of lutein have been reported in earlier studies [13]. Moreover, an unbalanced distribution of spermatogenic cells, vacuolation and significant damage to the seminiferous epithelium were among the unfavourable testicular architectural abnormalities shown by our histological analysis of the testis in the rats exposed to doxorubicin. Furthermore, testicular fibrosis was also brought on by increased collagen and extracellular metrix deposition. Remarkably, lutein decreased fibrosis and attenuated these histological abnormalities thereby maintaining reproductive health which was consistent with earlier studies [45–48].

## Conclusions

**Fig. 10:**
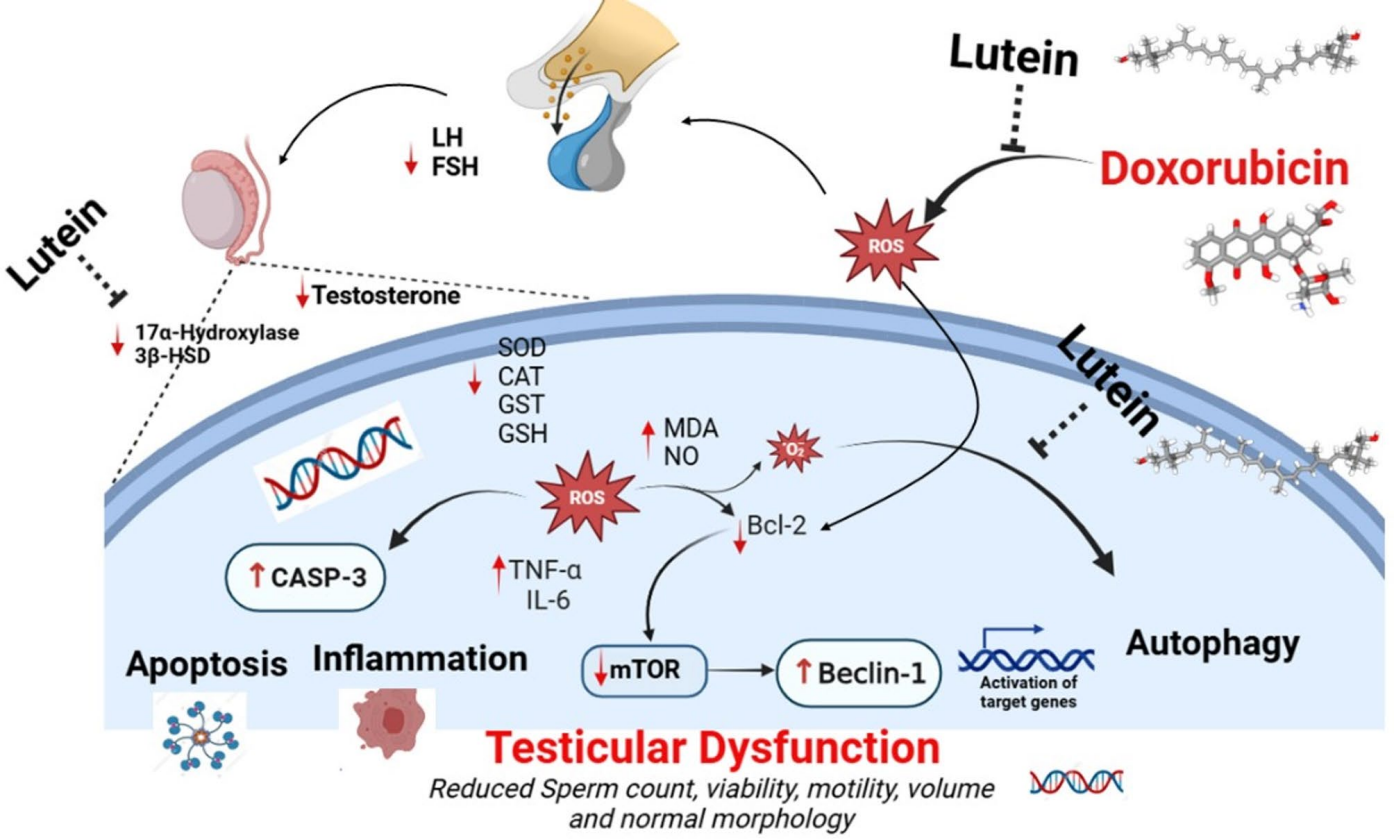
Graphical illustration of preventive mechanism of action of lutein

Finally, the results of the study validate lutein’s exceptional capacity to guard against certain doxorubicin-associated reproductive health consequences. While all of this is going on, lutein exhibits a plethora of other properties, such as anti-inflammatory, anti-apoptotic, antioxidant, and hormone balance maintenance. It also enhances spermatogenesis and helps to restore mTOR, Beclin-1, and other signalling pathways that are essential for the best possible functioning of cells. The probable mechanism of action of lutein can be attributed to its ability to suppress oxidative stress leading to attenuation of pro-inflammatory reactions, apoptotic activities and modulation of beclin-1/mTOR activities in the testes (Fig. 10). It also acts on the gonadotrophs to influence steroidogenesis and spermatogenesis. However, our team is currently working hard to elucidate these processes using modern molecular techniques. Meanwhile, it will be unjust to deny the scientific community, biochemical basis of our present investigation.

## Data Availability

All the data associated with this study has been included in this manuscript.

## Abbreviations

BCL-2: B-cell lymphoma factor 2
DOX: Doxorubicin
FSH: Follicle stimulating hormone
GSH: Glutathione
GST: Glutathione S-transferase
IL-6: Interleukin-6
LH: Luteinizing hormone
LUT: Lutein
MDA: Malondialdehyde
mTOR: Mammalian target of rapamycin
SOD: Superoxide dismutase
TNF-α: Tumour necrosis factor alpha
